# High-Fat Diet Increases Amylin Accumulation in the Hippocampus and Accelerates Brain Aging in hIAPP Transgenic Mice

**DOI:** 10.3389/fnagi.2019.00225

**Published:** 2019-08-27

**Authors:** Xiao-Xia Xi, Jing Sun, Hai-Chao Chen, An-Di Chen, Li-Ping Gao, Jie Yin, Yu-Hong Jing

**Affiliations:** ^1^Center of Experimental Animal, School of Basic Medical Sciences, Lanzhou University, Lanzhou, China; ^2^School of Basic Medical Sciences, Institute of Anatomy and Histology & Embryology, Neuroscience, Lanzhou University, Lanzhou, China; ^3^School of Basic Medical Sciences, Institute of Biochemistry and Molecular Biology, Lanzhou University, Lanzhou, China; ^4^Key Laboratory of Preclinical Study for New Drugs of Gansu Province, School of Basic Medical Sciences, Lanzhou University, Lanzhou, China

**Keywords:** human islet amyloid polypeptide, high-fat diet, hippocampus, cognition, brain aging

## Abstract

The accumulation of human islet amyloid polypeptide (hIAPP) in pancreatic islets under induction by a high-fat diet plays a critical role in the development of type-2 diabetes mellitus (T2DM). T2DM is a risk factor of late-onset Alzheimer’s disease (AD). Nevertheless, whether hIAPP in combination with hyperlipidemia may lead to AD-like pathological changes in the brain remains unclear. hIAPP transgenic mice were fed with a high-fat diet for 6 or 12 months to establish the T2DM model. The accumulation of amylin, the numbers of Fluoro-Jade C (FJC)-positive and β-gal positive cells, and the deposition level of Aβ42 in the hippocampi of the transgenic mice were detected by using brain sections. Cytoplasmic and membrane proteins were extracted from the hippocampi of the transgenic mice, and the ratio of membrane GLUT4 expression to cytoplasmic GLUT4 expression was measured through Western blot analysis. Changes in the cognitive functions of hIAPP transgenic mice after 12 months of feeding with a high-fat diet were evaluated. hIAPP transgenic mice fed with a high-fat diet for 6 or 12 months showed elevated blood glucose levels and insulin resistance; increased amylin accumulation, number of FJC-positive and β-gal positive cells, and Aβ42 deposition in the hippocampi; and reduced membrane GLUT4 expression levels. hIAPP transgenic mice fed with a high-fat diet for 12 months showed reductions in social cognitive ability and passive learning ability. A high-fat diet increased amylin accumulation in the hippocampi of hIAPP transgenic mice, which presented AD-like pathology and behavior characterized by neural degeneration, brain aging, Aβ42 deposition, and impaired glucose utilization and cognition.

## Introduction

Increasing evidence suggests that type-2 diabetes mellitus (T2DM) is characterized by aberrant metabolism that affects not only the peripheral organs and causes diabetic cardiovascular disease, diabetic retinopathy, diabetic nephropathy but also the brain (Forbes and Cooper, 2013; Wong et al., 2016; Benameur et al., 2019; Zubair and Ahmad, 2019). The effects of T2DM on the brain cause diabetic encephalopathy (Liu et al., 2018). Epidemiological data show that the risk of Alzheimer’s disease (AD) in patients with T2DM is two to five times higher than that in patients without T2DM. This association indicates that T2DM may be involved in the pathogenesis of neurodegenerative diseases *via* multiple aspects (Klimova et al., 2018; Moran et al., 2019). Progressive β-cell failure and insulin resistance are the most important pathological features of T2DM (DeFronzo et al., 2015). Underlying pancreatic islet defects may increase glucose tolerance by contributing to the inability of β-cells to compensate for increased insulin demand (Kowalski and Bruce, 2014). The cause of this β-cell dysfunction is unknown and is likely associated with genetic and environmental factors. One of these environmental factors is increased dietary fat, which has been associated with obesity and insulin resistance. Clinical and animal experimental studies have shown that hyperlipidemia leads to the development of insulin resistance (Li et al., 2018; Zheng et al., 2018; Feng et al., 2019). The vast majority of subjects with T2DM exhibit islet amyloid deposition in their pancreatic islets in addition to β-cell secretory defects (Weise et al., 2010; Guardado-Mendoza et al., 2017; Xin et al., 2017).

Amyloids mainly comprise islet amyloid polypeptide (IAPP). Amyloid deposition in islets is a typical molecular pathological feature of T2DM (Hull et al., 2004). IAPP is produced by islet β-cells and co-secreted with insulin (Kajava et al., 2005). The physiological function of IAPP remains unclear and may participate in the regulation of gastrointestinal motility (Cooper et al., 1988). IAPP is not implicated in the pathology of islet injury under physiological conditions. Increased secretion and IAPP misfolding are the initial factors of amyloid deposition (Clark et al., 1988). The ability of IAPP to induce amyloid deposition is species-specific. Although IAPP is highly conserved, its sequences show differences across species. For example, human IAPP (hIAPP) differs from rodent IAPP (rIAPP) by six amino acid residues. The structure of primate-derived IAPP is more similar to that of feline-derived IAPP than that to hIAPP. The probability of hIAPP deposition is based on the specific molecular structure of hIAPP (Khemtemourian et al., 2008; Guardado-Mendoza et al., 2009). IAPP deposition involves three steps. Linear IAPP initially forms an α-helix structure before forming a β-fold structure. Subsequently, it forms an oligomer, a polymer, and a fiber structure before finally forming nondegradable amyloid deposits. Amino acid (aa) residues 1–19 in the amino terminal of IAPP are the key regions for α-helix structure formation through disulfide bonding (Sasahara et al., 2014). hIAPP is more likely to form α-helix structures than rIAPP because aa18 is histidine in hIAPP and is arginine in rIAPP. The high susceptibility of histidine to protonation may be the structural basis for α-helix formation by hIAPP (Chakraborty et al., 2013).

IAPP can cross the blood-brain barrier (BBB) and is found in Aβ deposits in transgenic mice with AD (Banks et al., 1995; Chaitanya et al., 2011). IAPP and Aβ exhibit an overall aa sequence identity of 25% (O’Nuallain et al., 2004). Two regions of Aβ (i.e., aa 11–21 and aa 23–37) with high binding affinity for IAPP and two analogous regions on IAPP (aa 8–20 and aa 21–37) with corresponding affinity for Aβ have been identified. These binding zones include residues that are important for self-assembly (Andreetto et al., 2010). These pieces of evidence suggest that diabetic encephalopathy partly contributes to intensive IAPP generation and amyloid formation during T2DM development.

Although the pathological mechanisms of AD and T2DM share common factors, such as age, aberrant protein conformation and aggregation, and abnormal glucose utilization, their molecular mechanisms remain unclear. The purpose of our study was to explore the roles of amylin in the brains of hIAPP transgenic mice fed with a high-fat diet. We explored whether hIAPP transgenic mice exhibited AD-like pathological characteristics.

## Materials and Methods

### Reagents

Rabbit polyclonal anti-Aβ42 antibody was procured from Millipore (Millipore, Cat# AB5078P, CA, USA). Rabbit polyclonal anti-amylin antibody was obtained from Boster (Boster, Cat#A00414, Wuhan, China). Mouse monoclonal anti-Glu4 (Santa Cruz, Cat# sc-53566, CA, USA) and Na^+^/K^+^-ATPases antibodies were purchased from Santa Cruz (Santa Cruz, Cat# sc-58628, CA, USA). Rabbit polyclonal anti-GAPDH antibody was purchased from ImmunoWay (ImmunoWay, Cat# YM3445, TX, USA). β-Galactosidase staining kits (Beyotime, Cat# C0602, Haimen, China) and membrane and cytosol protein extraction kits were purchased from Beyotime (Beyotime, Cat# P0027, Haimen, China).

### Animals

Transgenic hIAPP male mice with a FVB/N background (Jackson Laboratory, Cat# 008232) were maintained by mating heterozygous transgenic mice with FVB/N mice. Transgenic mice were identified through the PCR analysis of total DNA by using specific primers (transgenic: forward, 5′-TGAAAAAGTCCACTAATTAAAACCA-3′; reverse, 5′-CTAACAACCCTTTCTCTCAAGGT-3′. Nontransgenic: forward, 5′-GATTTGAGGGACGCTGTGG-3′; reverse, 5′-GTGGCAGTGT TGCAT TTCC-3′). Mice were bred and housed in an animal housing facility and maintained in air-conditioned rooms at 20°C–22°C with a light period of 12 h. All mice were given feed and water *ad libitum*. The regular chow contained 4,500 kcal/kg and was composed of 22.5% protein and 4.8% fat (Hope Farms, Woerden, Netherlands). The high-fat chow contained 5,600 kcal/kg and was composed of 20.8% protein and 36.0% fat (30.0% cocoa oil, 6.0% corn oil; Hope Farms). Mice were fed with regular or high-fat chow for 6 or 12 months. This study was carried out in accordance with the recommendations of the ethical treatment of laboratory animals of the Ministry of Science and Technology of the People’s Republic of China. All animal experiments were approved by the Experimental Animal Ethics Committee of Lanzhou University.

### Glucose Tolerance Test

The mice were fasted overnight prior to the glucose tolerance test (GTT) after 6 and 12 months of treatment. The mice were intraperitoneally injected with glucose (1 g/kg body weight dissolved in sterile saline), and blood was collected from the tail vein. Glucose levels were measured at 0, 30, 60, 90, and 120 min after injection by using OneTouch Ultra glucometer (SANNUO, Changsha, China).

### Insulin Tolerance Test

The insulin tolerance test (ITT) was conducted 2 days after the GTT experiment. The mice were fasted overnight and intraperitoneally injected with insulin (0.75 IU/kg body weight). Blood glucose was measured immediately before insulin administration and at 30, 60, 90, and 120 min after injection.

### Behavioral Tests

After 12 months of treatment, the mice in each group were subjected to the open-field test, three-chamber social test, and step-down test in accordance with previously described methods.

### Open-Field Test

The open-field test was performed in a black box with dimensions of 60 cm × 60 cm × 25 cm. The central area was defined with dimensions of 20 cm × 20 cm. The movement of each mouse in the box was recorded for 5 min, and moving time and moving distance were analyzed by using a video-tracking system (TM-vision, Chengdu Techman Software Company, Limited, Chengdu, China).

### Three-Chamber Social Test

Social preference was initially tested in accordance with previous methods. A familiar conspecific male mouse housed in a small cage (8 cm × 8 cm × 8 cm) was placed on one side of three chambers, and an empty cage was placed on another side. The number of times the mouse stayed in each chamber over 10 min was recorded. The same mouse was placed on one side of the three chambers after 6 h, and an unfamiliar male mouse was placed on another side of the chamber. The number of times the mouse stayed in each chamber over 10 min was recorded.

### Step-Down Test

The instrument comprised a test box (10 × 10 cm). A safe round platform (diameter: 4 cm) was located in a corner of the test box. The mice were placed in the test box for 60 s during the training period to allow them to adapt to the environment. Electrical stimulation was maintained at the bottom of the box for 300 s at 32 V. After 48 h, the mice were placed in the box, and 32 V of electrical stimulation was provided at the bottom of the box. The number of times the mice stayed in the platform and the error response in the test box during 300 s was recorded (ST-120, Chengdu Techman Software Company, Limited, Chengdu, China).

### Preparation of Brain Sections

The mice were anesthetized with phenobarbitone (350 mg/kg, i.p.) and perfused with 4% paraformaldehyde through the heart after 6 or 12 months of treatment. Brains were collected, postfixed in 4% PA for 24 h, and immersed in 20% and 30% sucrose until they sank completely. Sections with thicknesses of 20 μm were obtained by using a cryostat microtome. All brain sections were stored in frozen protective solution for later use.

### Fluoro-Jade C Staining

Fluoro-Jade C (FJC) staining and imaging analysis were performed as previously described. In brief, brain sections were dipped in 80% ethanol solution containing 1% sodium hydroxide for 5 min; in 70% ethanol for 2 min; and then in 0.06% potassium permanganate for 10 min. Sections were rinsed with distilled water and then incubated with 0.0004% FJC in 0.1% acetic acid for 20 min. FJC staining was observed under a fluorescent microscope with excitation at 480 nm and emission at 525 nm. The images were acquired under 20× magnification, and FJC-positive cells in the hippocampal CA1 and CA3 regions were counted by using ImageJ software.

### β-gal Staining

Brain sections were washed with 0.01 M PBS, fixed with 4% paraformaldehyde for 15 min and washed with 0.01 M PBS. Sections were transferred into a 1.5 mL tube and incubated with working staining solution at 37°C for 6 h. Sections were mounted and counterstained with neutral red. The images of the hippocampal CA1 and CA3 regions were captured under microscopy, and positive cells were counted.

### Immunohistochemistry

Brain sections were washed with 0.01 M PBS, incubated with 10% goat serum at 37°C for 1 h, and incubated with Aβ42 antibody (1:200) at 4°C overnight. Sections were rinsed with 0.01 M PBS and incubated with the corresponding second antibody (1:200) at room temperature for 1 h. Sections were rinsed with 0.01 M PBS and incubated with strep–avidin–AP (1:200) at room temperature for 2 h, developed with BCIP chromogenic reagent for 10 min, and washed with 0.01 M TBST. Sections were mounted and counterstained for observation under microscope. The presence of positive particles in the hippocampal CA1 region and prefrontal cortex (PFC) was analyzed.

### Amylin Immunofluorescence

Brain sections were selected and rinsed with 0.01 M PBS, incubated with 10% goat serum at 37°C for 45 min, and incubated with sheep antirabbit amylin antibody (1:200) at 4°C overnight. Sections were rinsed with 0.01 M PBS and incubated with goat antirabbit IgG coupling with Dylight-594 (1:200) at 37°C for 2 h under dark. Sections were rinsed with 0.01 M PBS and counterstained with DAPI. Amylin-positive particles were observed by using a fluorescence microscope.

### Counting of Immunostaining

Cell counting was performed in a double-blinded fashion. Six coronal sections (i.e., 20 μm thickness at 200 μm intervals) at the bregma level of −1.06 to −2.46 mm (dorsal hippocampus) or 2.8 mm to 1.4 mm PFC were obtained to identify FJC, β-gal, Aβ42, and amylin through immunohistochemistry. Positive immunostaining in each section of the hippocampus or PFC were counted as previously described. In brief, the total positive number was estimated in accordance with the following formula: [(S1 + S2)/2 + (S2 + S3)/2 + (S3 + S4)/2 + (S4 + S5)/2 + (S5 + S6)/2] × 10, where S1–S6 represent the positive number in sections 1–6, respectively. The coefficient 10 reflects the selection of one section (20 μm thickness) from 10 serial coronal sections (200 μm intervals) for staining. Six sections from each animal were selected and stained with FJC, β-gal, Aβ42, and amylin.

### Western Blot Analysis

Cytoplasmic and cytomembrane proteins were extracted from the hippocampi of mice. Protein content was quantified through the Bradford method. Proteins (50 μg) were fractionated on 10% sodium dodecyl sulfate polyacrylamide gel for electrophoresis and transferred to polyvinylidene fluoride membranes. The membranes were blotted with Glut4 antibody (Santa Cruz, CA, USA; 1:500), GAPDH antibody (ImmunoWay, TX, USA; 1:500), and Na^+^/K^+^-ATPase antibody (Santa Cruz, CA, USA; 1:500) and incubated with horseradish peroxidase-conjugated second antibody (1:5,000). Immunoreactive bands were visualized using enhanced chemiluminescence.

### Statistical Analysis

Data were expressed as mean ± SEM and processed using SPSS17.0. *T*-test of independent samples was performed to compare two groups, and one-way ANOVA was used for comparison among multiple groups. *p* < 0.05 was considered statistically significant.

## Results

### Effects of a High-Fat Diet on the Blood Biochemical Parameters of hIAPP^−/+^ Mice

**Figure 1:**
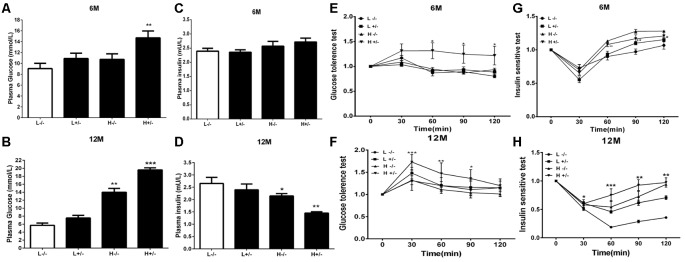
Blood biochemistry parameters of human islet amyloid polypeptide (hIAPP^−/−^) or hIAPP^−/+^ mice fed with a high/low-fat diet for 6 or 12 months. **(A)** Plasma glucose content at 6 months after treatment. **(B)** Plasma glucose content at 12 months after treatment. **(C)** Plasma insulin content at 6 months after treatment. **(D)** Plasma insulin content at 12 months after treatment. **(E)** Results of the glucose tolerance test (GTT) at 6 months after treatment. **(F)** Results of the GTT at 12 months after treatment. **(G)** Results of the insulin sensitivity test at 6 months after treatment. **(H)** Results of the insulin sensitivity test at 12 months after treatment. L^−/−^ denotes hIAPP^−/−^ mice on a low-fat diet; L^+/–^ denotes hIAPP^+/–^ mice on a low-fat diet; H^−/−^ denotes hIAPP^−/−^ mice on a high-fat diet; H^+/–^ denotes hIAPP^+/–^ mice on a high-fat diet. *n* = 12, *p < 0.05, **p < 0.01, ***p < 0.001 compared with the L^−/−^ group.

The blood glucose levels of hIAPP^−/+^ mice fed with a high-fat diet for 6 months significantly increased (*p* < 0.01, Figure 1A). Serum insulin level remained unchanged (Figure 1C), glucose tolerance increased (*p* < 0.05, Figure 1E), and insulin sensitivity decreased (*p* < 0.05, *p* < 0.01, Figure 1G) in IAPP^−/−^ mice fed with a low-fat diet relative to those in hIAPP^−/−^ mice fed with a low-fat diet. hIAPP^−/+^ mice fed with a high-fat diet for 12 months showed significantly elevated blood glucose levels (*p* < 0.001, Figure 1B), decreased serum insulin levels (*p* < 0.01, Figure 1D), enhanced glucose tolerance (*p* < 0.05, *p* < 0.01, Figure 1F), and reduced insulin sensitivity (*p* < 0.01, *p* < 0.001, Figure 1H) when compared with hIAPP^−/−^ mice fed with a low-fat diet. Blood glucose levels increased (*p* < 0.01, Figure 1B) and serum insulin level decreased (*p* < 0.05, Figure 1D) in hIAPP^−/−^ mice fed with a high-fat diet compared with those in hIAPP^−/−^ mice fed with a low-fat diet. These results indicate that hIAPP^−/+^ mice on a high-fat diet exhibited increased blood glucose, decreased insulin levels, and enhanced insulin resistance in a time-dependent pattern.

### Effects of a High-Fat Diet on Amylin Deposition in the Hippocampi of hIAPP^−/+^ Mice

**Figure 2:**
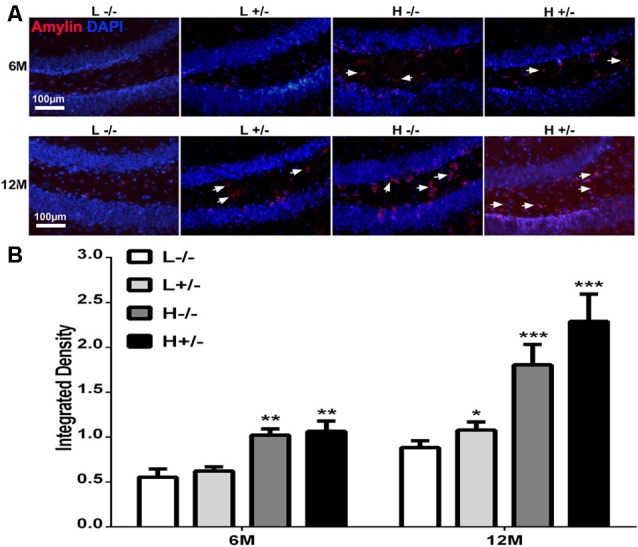
Amylin content in the hippocampi of hIAPP^−/−^ and hIAPP^+/–^ mice increased under a high-fat diet. **(A)** Representative images of amylin-positive particles in the DG of the hippocampus. White arrows indicate positive staining with the amylin antibody, and blue staining indicates the nucleus. **(B)** Six sections per mouse were stained, and positive particles were analyzed by using ImageJ software. L^−/−^ denotes hIAPP^−/−^ mice on a low-fat diet; L^+/–^ denotes hIAPP^+/–^ mice on a low-fat diet; H^−/−^ denotes hIAPP^−/−^ mice on a high-fat diet; H^+/–^ denotes hIAPP^+/–^ mice on a high-fat diet. *n* = 6, *p < 0.05, **p < 0.01, ***p < 0.001 compared with the L^−/−^ group.

Amylin deposition in the hippocampi of hIAPP^−/+^ mice fed with a high-fat diet for 6 months was significantly higher than that in hIAPP^−/−^ mice fed with a low-fat diet (*p* < 0.01, Figures 2A,B). Amylin deposition in the hippocampi of hIAPP^−/+^ mice fed with a high-fat diet for 12 months increased more significantly than that in the hippocampi of hIAPP^−/−^ mice fed with a low-fat diet (Figures 2A,B, *p* < 0.001). Amylin deposition was higher in hIAPP^−/−^ mice on a high-fat diet than in hIAPP^−/−^ mice on a low-fat diet (*p* < 0.001). These results suggest that high-fat diets induced IAPP (rIAPP and hIAPP) expression and increased amylin deposition in the hippocampus.

### Effects of a High-Fat Diet on Hippocampal Damage in hIAPP^−/+^ Mice

**Figure 3:**
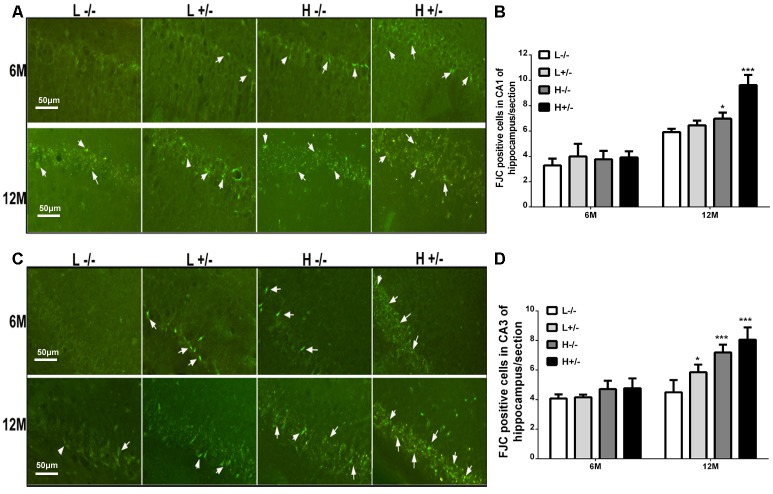
Neural degeneration in the hippocampi of hIAPP^−/−^ and hIAPP^−/+^ mice was accelerated by a high-fat diet.** (A)** Representative images of Fluoro-Jade C (FJC)-positive staining in the hippocampal CA1 regions of hIAPP^−/−^ and hIAPP^+/–^ mice fed with a high/low-fat diet for 6 or 12 months. White arrows indicate FJC-positive cells.** (B)** Six sections per mouse were stained, and semiquantitative analysis was performed using ImageJ software. **(C)** Representative images of FJC-positive staining in the hippocampal CA3 regions of hIAPP^−/−^ and hIAPP^+/–^ mice fed with a high/low-fat diet for 6 or 12 months. White arrows indicate FJC-positive cells. **(D)** Six sections per mouse were stained, and semiquantitative analysis was performed using ImageJ software. L^−/−^ denotes hIAPP^−/−^ mice on a low-fat diet; L^+/–^ denotes hIAPP^+/–^ mice on a low-fat diet; H^−/−^ denotes hIAPP^−/−^ mice on a high-fat diet; H^+/–^ denotes hIAPP^+/–^ mice on a high-fat diet. *n* = 6, *p < 0.05, ***p < 0.001 compared with the L^−/−^ group.

FJC staining was used to label damaged cells. The number of FJC-positive cells in the hippocampal CA1 and CA3 regions of hIAPP^−/+^ mice fed with a high-fat diet for 6 months was not significantly different from that in the hippocampal CA1 and CA3 regions of hIAPP^−/−^ mice (Figures 3A–D). The number of FJC-positive cells in the hippocampal CA1 and CA3 regions of hIAPP^−/+^ mice fed with a high-fat diet for 12 months increased significantly compared with that in the hippocampal CA1 and CA3 regions of hIAPP^−/−^ mice (*p* < 0.001, Figures 3A–D).

### Effects of a High-Fat Diet on Nerve Cell Aging in Hippocampus of hIAPP^−/+^ Mice

**Figure 4:**
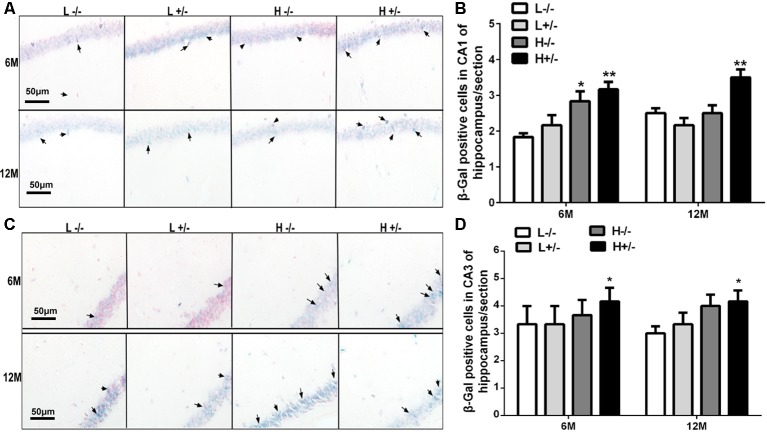
Nerve cell aging in the hippocampi of hIAPP^−/−^ and hIAPP^−/+^ mice was accelerated by a high-fat diet. **(A)** Representative images of β-gal-positive staining in the hippocampal CA1 regions of hIAPP^−/−^ and hIAPP^+/–^ mice fed with a high/low-fat diet for 6 or 12 months. Black arrows indicate β-gal-positive cells. **(B)** Six sections per mouse were stained, and semiquantitative analysis was performed using ImageJ software. **(C)** Representative images of β-gal-positive staining in the hippocampal CA3 regions of hIAPP^−/−^ and hIAPP^+/–^ mice treated with a high/low-fat diet for 6 or 12 months. Black arrows indicate β-gal-positive cells. **(D)** Six sections per mouse were stained, and semiquantitative analysis was performed using ImageJ software. L^−/−^ denotes hIAPP^−/−^ mice on a low-fat diet; L^+/–^ denotes hIAPP^+/–^ mice on a low-fat diet; H^−/−^ denotes hIAPP^−/−^ mice on a high-fat diet; H^+/–^ denotes hIAPP^+/–^ mice on a high-fat diet. *n* = 6, *p < 0.05, **p < 0.01 compared with the L^−/−^ group.

Aging cells were labeled through β-galactosidase staining. The number of β-gal-positive cells in the hippocampal CA1 region of hIAPP^−/+^ mice fed with a high-fat diet for 6 or 12 months had significantly increased compared with that in hIAPP^−/−^ mice fed with a low-fat diet (*p* < 0.01, Figures 4A,B). The same changes can be observed in the CA3 region of the hippocampus (*p* < 0.05, Figures 4C,D).

### Effects of a High-Fat Diet on Aβ42 Deposition in the Hippocampus and PFC in hIAPP^−/+^ Mice

**Figure 5:**
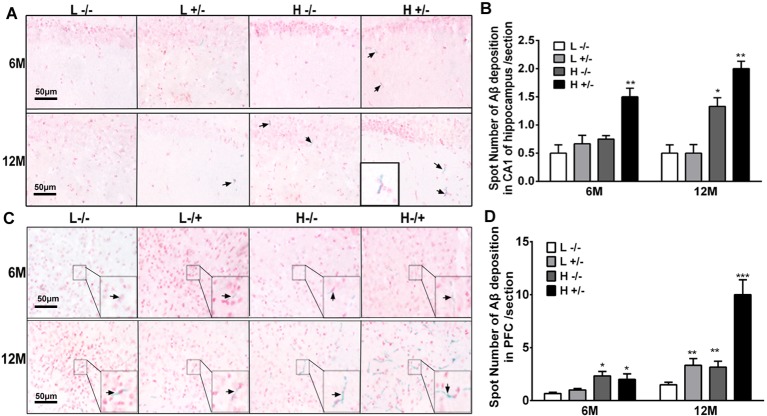
Aβ42 deposition in the hippocampi and prefrontal cortexes of hIAPP^−/−^ and hIAPP^−/+^ mice were enhanced by a high-fat diet. **(A)** Representative images of Aβ42-positive staining in the hippocampal CA1 regions of hIAPP^−/−^ and hIAPP^+/–^ mice treated with a high/low-fat diet for 6 or 12 months. Black arrows indicate Aβ42-positive particles. **(B)** Six sections per mouse were stained, and semiquantitative analysis was performed using ImageJ software. **(C)** Representative images of Aβ42-positive staining in the prefrontal cortexes of hIAPP^−/−^ and hIAPP^+/–^ mice treated with a high/low-fat diet for 6 or 12 months. Black arrows indicate Aβ42-positive particles. **(D)** Six sections per mouse were stained, and semiquantitative analysis was performed using imageJ software. L^−/−^ denotes hIAPP^−/−^ mice on a low-fat diet; L^+/–^ denotes hIAPP^+/–^ mice on a low-fat diet; H^−/−^ denotes hIAPP^−/−^ mice on a high-fat diet; H^+/–^ denotes hIAPP^+/–^ mice on a high-fat diet. *n* = 6, *p < 0.05, **p < 0.01, ***p < 0.001 compared with the L^−/−^ group.

Aβ42 deposition in the hippocampal CA1 region was observed by using immunohistochemistry. The amount of Aβ42 in the hippocampi of hIAPP^−/+^ mice fed with a high-fat diet for 6 or 12 months significantly increased compared with that in the hippocampi of hIAPP^−/−^ mice on a low-fat diet (*p* < 0.05, *p* < 0.01, Figures 5A,B; Supplementary Figure S2). The amount of Aβ42 in the hippocampal CA1 region of hIAPP^−/+^ mice on a low-fat diet for 12 months also increased significantly compared with that in hIAPP^−/−^ mice on a low-fat diet (*p* < 0.05, Figures 5A,B). The same changes can be observed in the PFC region (Figures 5C,D). The amount of Aβ42 in the PFC region in hIAPP^−/+^ mice on a low-fat diet was higher than that in hIAPP^−/−^ mice on a low-fat diet (*p* < 0.01, Figures 5C,D). This result suggests that the high expression of hIAPP alone is an independent risk factor for Aβ42 deposition in PFC.

### Effects of a High-Fat Diet on GLUT4 Expression in the Hippocampi of hIAPP^−/+^ Mice

**Figure 6:**
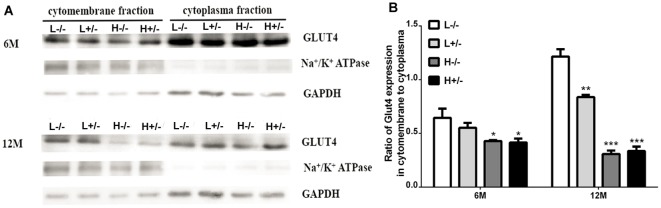
GLUT4 expression in the membrane fractions of the hippocampi of hIAPP^−/−^ and hIAPP^+/–^ mice was reduced by a high-fat diet. **(A)** Representative images of Western blot analysis. **(B)** Quantitative analysis was performed on the basis of gray density by using ImageJ software. L^−/−^ denotes hIAPP^−/−^ mice on a low-fat diet; L^+/–^ denotes hIAPP^+/–^ mice on a low-fat diet; H^−/−^ denotes hIAPP^−/−^ mice on a high-fat diet; H^+/–^ denotes hIAPP^+/–^ mice on a high-fat diet. *n* = 6, *p < 0.05, **p < 0.01, ***p < 0.001 compared with the L^−/−^ group.

Insulin-dependent glucose transport involves GLUT4 expression on the cell membrane. GLUT4 expression in the hippocampal membrane and cytoplasm was detected through Western blot analysis. GLUT4 expression on the cell membrane in hIAPP^−/+^ mice fed with a high-fat diet for 6 and 12 months significantly decreased relative to that in hIAPP^−/−^ mice fed with a low-fat diet (*p* < 0.05, *p* < 0.001, Figures 6A,B). The reduction in the expression levels of GLUT4 in the cell membrane fraction of hIAPP^−/−^ mice fed with a high-fat diet for 6 or 12 months relative to that in hIAPP^−/−^ mice fed with a low-fat diet (*p* < 0.05, *p* < 0.001, Figures 6A,B) suggests that a high-fat diet can independently reduce GLUT4 expression on cell membranes.

### Effects of a High-Fat Diet on the Learning and Memory of hIAPP^−/+^ Mice

**Figure 7:**
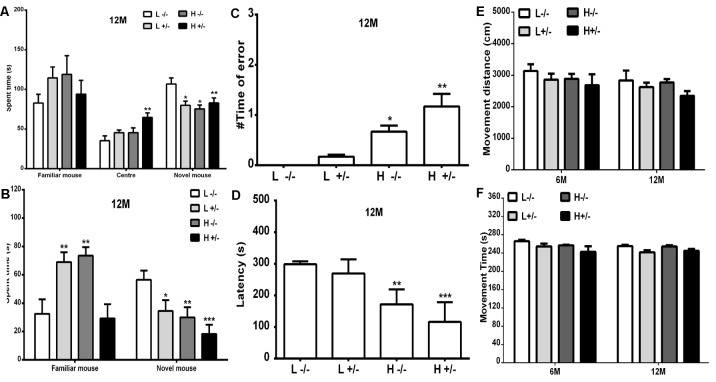
Cognitive ability of hIAPP^−/−^ and hIAPP^+/–^ mice was impaired by a high-fat diet. **(A,B)** Social learning ability of hIAPP^−/−^ and hIAPP^+/–^ mice fed with a high/low-fat diet for 12 months was tested through the 3-chamber social test. **(A)** Time spent in different chambers was analyzed through the three-chamber social test **(A)**. **(B)** Time spent in social areas occupied by familial mouse and novel mouse was analyzed **(B)**. **(C,D)** Step-down test was used to evaluate the passive learning ability of hIAPP^−/−^ and hIAPP^+/–^ mice treated with a high/low-fat diet for 12 months. Times of error in step-down were analyzed **(C)**. Time spent on the safe platform was analyzed **(D)**. **(E,F)** Open-field test was performed to evaluate the movement function of hIAPP^−/−^ and hIAPP^+/–^ mice fed with a high/low-fat diet for 6 and 12 months. The movement distance **(E)** and movement time **(F)** were analyzed. L^−/−^ denotes hIAPP^−/−^ mice on a low-fat diet; L^+/–^ denotes hIAPP^+/–^ mice on a low-fat diet; H^−/−^ denotes hIAPP^−/−^ mice on a high-fat diet; H^+/–^ denotes hIAPP^+/–^ mice on a high-fat diet. *n* = 12, *p < 0.05, **p < 0.01, ***p < 0.001 compared with the L^−/−^ group.

The changes in the social learning abilities of mice were observed through the three-chamber social test. The time spent in an area by a novel hIAPP^−/+^ mouse fed with a high- or a low-fat diet was shorter than that spent by a novel hIAPP^−/−^ mouse fed with a low-fat diet (*p* < 0.05, *p* < 0.01, Figure 7A). The time spent in the middle area by hIAPP^−/+^ mice on a high-fat diet was longer than that spent by hIAPP^−/−^ mice on a low-fat diet (*p* < 0.01, Figure 7A). The time spent in the middle area was deducted to analyze the exploration and recognition ability of the mice. The time spent in an area and the time spent in social exploration by a novel hIAPP^−/+^ mouse on a high-fat diet were significantly lower than those spent by a hIAPP^−/−^ mouse on a low-fat diet (*p* < 0.01, Figure 7B). Several brain regions, including the hippocampus, olfactory bulb, and hypothalamus, are related to the regulation of social exploration and recognition. The step-down test was also applied to examine the passive learning ability of the mice. The number of errors in finding the safe platform shown by hIAPP^−/+^ mice on a high-fat diet was higher than that shown by hIAPP^−/−^ mice on a low-fat diet (*p* < 0.01, Figure 7C). The time latency to find the safe platform shown by hIAPP^−/+^ mice on a high-fat diet was higher than that shown by hIAPP^−/−^ mice on a low-fat diet (*p* < 0.001, Figure 7D). The passive learning ability of hIAPP^−/+^mice on a high-fat diet was impaired relative to that of hIAPP^−/−^ mice on a low-fat diet (*p* < 0.05, *p* < 0.01, Figures 7C,D). The effects of a high-fat diet on the motor and spatial exploration abilities of hIAPP^−/+^ mice were evaluated through the open-field test. Moving distance and time did not significantly differ between groups (Figures 7E,F).

## Discussion

The pathogenesis of T2DM and AD share numerous similarities. Specifically, both diseases are age-dependent and feature abnormal protein aggregation (Karki et al., 2017; Paul et al., 2018). IAPP is deposited in the pancreatic islets of patients with T2DM, and Aβ is deposited in the brains of patients with AD. IAPP deposition results in β-cell loss and reduces insulin secretion. Aβ deposition impairs cognitive function by causing neuronal loss in specific brain regions, such as the hippocampus and PFC (Shankar et al., 2008; Bailey et al., 2011). Given the structural similarity between hIAPP and Aβ and the ability of IAPP to cross the BBB, we attempted to explore whether islet-derived hIAPP, a key molecule implicated in T2DM, results in AD-like pathology in the brain. Our results show that hIAPP^−/+^ transgenic mice fed with a high-fat diet for 12 months exhibited drastically increased neural degeneration, amylin accumulation in the hippocampi, Aβ42 deposition in the hippocampi and PFC, and accelerated hippocampal aging. The efficiency of glucose utilization in the hippocampi of hIAPP^−/+^ mice fed with a high-fat diet for 6 or 12 months decreased. The active and passive learning abilities and memory of hIAPP^−/+^ mice fed with a high-fat diet for 12 months were impaired. These results implicate T2DM induction in brain injury characterized by AD-like pathology, such as neural degeneration, Aβ deposition, energy deficit, brain aging, and cognitive impairment, in hIAPP^−/+^ transgenic mice on a high-fat diet. From the present data, it is speculated that high-fat diet can induce the continuous high expression of hIAPP which cross the BBB into the central nervous system in hIAPP transgenic mice. High-fat diet also affects the metabolism of Aβ42 in the brain, leading to the accumulation of Aβ42. The interaction between amylin and Aβ42 in hippocampus aggravates the degeneration of nerve cells (the increase of FJC positive cells) and the aging of nerve cells (the increase of β-gal positive cells).

Various methods have failed to provide evidence showing that amylin can be generated from the brain (Jackson et al., 2013; Srodulski et al., 2014). Pro-IAPP mRNA was not found in brain tissue. Amylin can enter the central nervous system through the BBB (Banks and Kastin, 1998). Amylin in the brain may have originated from the periphery, but whether cerebral Aβ contains amylin remains controversial. Some studies have shown that IAPP and Aβ colocalize in cerebral Aβ plaques, whereas Aβ is absent in pancreatic islet amyloid deposits (Oskarsson et al., 2015). Other studies have suggested that the microscopic events of amyloid species formation by IAPP are drastically different from those by Aβ42 (Hoffmann et al., 2018). The absence of oligomers in the lag phase of IAPP fibril formation suggests that these species are too unstable to give rise to large populations (Seeliger et al., 2012). Using the amylin antibody to distinguish amylin in Aβ deposits and rule out the possibility of amylin antibody binding to Aβ itself is difficult. However, a relationship between amylin and Aβ can be partially established if the increase in peripheral amylin results in the simultaneous increase of amylin and Aβ in the brain. The considerable increase in amylin levels in the hippocampi of hIAPP transgenic mice suggests that amylin with a peripheral origin was deposited in the hippocampus. This finding is consistent with the increase in Aβ42 level, the increase in FJC-positive cells (cell degeneration), and the increase in β-gal-positive cells (cell aging) in the hippocampi of hIAPP transgenic mice. These results suggest that amylin production by islets is a risk factor for the aggravation of AD-like pathology in the hippocampus. We could not define the hIAPP and rIAPP contents of amylin because no specific antibody can accurately distinguish between hIAPP and rIAPP. The drastic increase in the mRNA level of hIAPP in pancreatic islets in hIAPP transgenic mice on a high-fat diet suggests that the proportion of hIAPP peptides in peripheral amylin had considerably increased. Additionally, our results showed the increase of amylin level in hippocampus was synchronized with the increase of Aβ42. In our experiment, the hIAPP transgenic mice were used, and the endogenous IAPP of mice (rIAPP) is also expressed with high fat diet, so the amylin in the brain may be composed of two parts, one is hIAPP, the other is rIAPP. Considering that the animal model was characterized by the high expression of hIAPP, it was possible that hIAPP promoted the accumulation of Aβ42.

In this experiment, mice were fed with a high-fat diet, because the previous study suggested IAPP production is inducible by glucose or lipid (Qi et al., 2010; Visa et al., 2015). We found that amylin also increased in hIAPP^−/−^ mice on a high-fat diet. This result suggests that amylin in the brain was also derived from rIAPP. Consistently, Aβ42 levels in the hippocampus also increased in hIAPP^−/+^ and hIAPP^−/−^ mice fed with a high-fat diet for 12 months. These data show that amylin and Aβ in the hippocampus play interactive role (Jackson et al., 2013). Glucose is the major energy source of brain metabolism (Liguori et al., 2019). Impairments in brain glucose uptake and utilization have been detected in the preclinical stages of AD (Zhou et al., 2018). Furthermore, the prospective Baltimore Longitudinal Study has shown that impairments in brain glucose uptake are correlated with the reduced expression of the GLUT3 glucose transporter and subsequent development of AD (An et al., 2018). Mechanistically, given that glucose uptake and utilization in the brain and neuronal cells are stimulated by insulin, insulin deficiency or insulin resistance could disturb energy metabolism and thus contribute to the pathogenesis of AD. We analyzed the hippocampal expression of the insulin-dependent glucose transporter GLUT4 given that insulin deficiency is the primary pathology of T2DM. The considerable increase in the expression of GLUT4 on the cell membrane in hIAPP^−/+^ mice fed with a high-fat diet for 6 or 12 months indicates reductions in glucose transport ability and impairments in glucose utilization. The dependence of nerve cells on glucose as an energy source is considerably higher than that of other cells and may indicate abnormal energy utilization in nerve cells in the early stage of T2DM and reflect a causal relationship between T2DM and AD (Chornenkyy et al., 2019).

Previous studies have shown that T2DM accelerates brain aging, which is the key factor in the pathogenesis of AD (Bangen et al., 2018; Ettcheto et al., 2018). We also evaluated brain aging and found that the number of aging cells in the hippocampi of hIAPP^−/+^ transgenic mice fed with a high-fat diet for 6 or 12 months had increased. This result indicates that T2DM status accelerated nerve cell aging. We used two behavioral paradigms to detect the changes in the learning and memory ability of mice to evaluate the cognitive changes that occur during diabetes. First, we examined active learning ability by using social recognition paradigms. Social recognition ability decreased in hIAPP^−/+^ transgenic mice fed with a high-fat diet for 12 months. We quantified the passive learning ability of mice through the step-down experiment. Passive learning and contextual memory ability were also impaired in hIAPP^−/+^ transgenic mice fed with a high-fat diet for 12 months. Cognitive impairment was consistent with the increase in amylin, Aβ42, and aging cells in the hippocampus.

This study was mainly limited by our inability to clarify whether cerebral Aβ deposits contain an amylin component [Aβ42 and amylin amyloid deposits both can stain with Thioflavin S (Supplementary Figure S1)] or accompany the decline in insulin signaling in the brains of hIAPP transgenic mice. These problems require resolution in future experiments. Another limitation is only male mice were used in our experiment. Considering that lipid metabolism and insulin sensitivity were regulated by estrogen (May et al., 2016; Morselli et al., 2018), and the body fat content of female mice was also higher than that of male mice, which may introduce more complex factors. Therefore, only male mice were used in this experiment. Although several issues remain to be clarified, we found that islet-derived hIAPP is implicated in brain aging, Aβ deposition, and reduced glucose utilization under high-lipid conditions. These effects impaired the cognitive ability of mice and resulted in AD-like pathology and behavior.

## Data Availability

The datasets generated for this study are available on request to the corresponding author.

## Ethics Statement

This study was carried out in accordance with the recommendations of the ethical treatment of laboratory animals of the Ministry of Science and Technology of the People’s Republic of China. All animal experiments were approved by the Experimental Animal Ethics Committee of Lanzhou University.

## Author Contributions

Y-HJ planned experiments, interpreted data, approved the version to be published and wrote the article. X-XX and JS performed most of the experiments and analyzed data. H-CC and A-DC participated in the animal experiment. L-PG and JY participated in acquisition of the study specimens. All authors read and approved the final article.

## Conflict of Interest Statement

The authors declare that the research was conducted in the absence of any commercial or financial relationships that could be construed as a potential conflict of interest.
