# Supplementary material for: High-Fat Diet Increases Amylin Accumulation in the Hippocampus and Accelerates Brain Aging in hIAPP Transgenic Mice
Source: Front Aging Neurosci. 2019 Aug 27;11:225. doi: 10.3389/fnagi.2019.00225 (PMC6718729; doi:10.3389/fnagi.2019.00225)
Supplement: Supplementary file 1 [file Image_1.pdf]

Supplemental Figures

Supplemental Figure 1

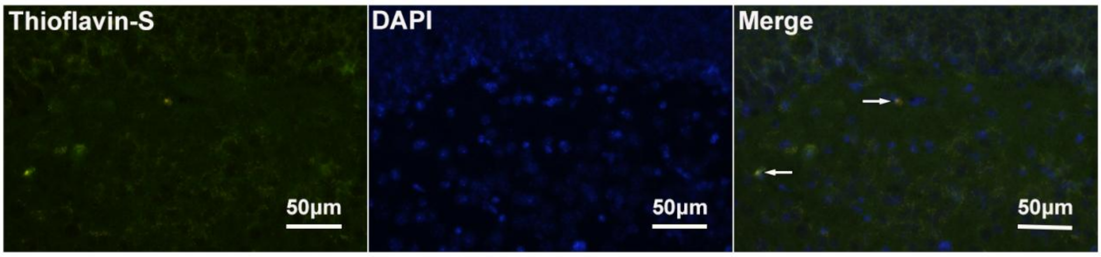

Figure S1. Thioflavin-S staining in hippocampus of hIAPP−/+ mice fed with a high-fat diet for 12 months. Representative images of Thioflavin-S positive staining in the hippocampal of hIAPP+/− mice fed with a high-fat diet for 12 months. Green staining indicate the Thioflavin-S positive staining, blue staining indicate the nucleus, White arrows indicate Thioflavin-S positive staining.

Supplemental Figure 2

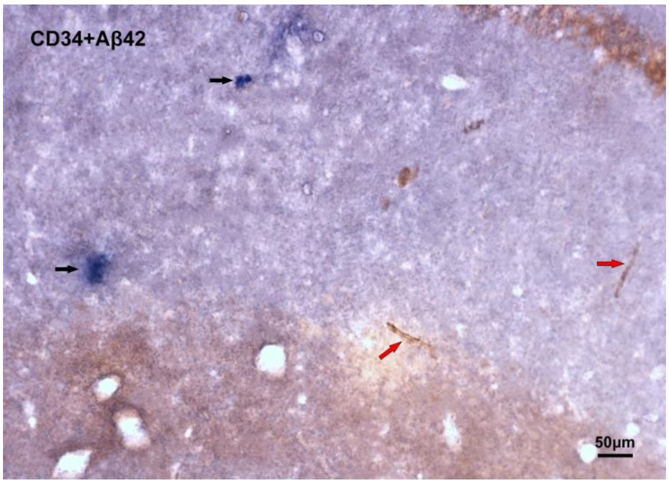

Figure S2. CD34 and Aβ42 immunohistochemical staining in hippocampus of hIAPP−/+ mice fed with a high-fat diet for 12 months. Representative images of CD34 (vascular endothelial cells) and Aβ42 positive staining in the hippocampal of hIAPP+/− mice fed with a high-fat diet for 12 months. Black arrows indicate Aβ42 positive staining, red arrows indicate CD34 positive staining.
